# Transcript Analysis of Zebrafish GLUT3 Genes, *slc2a3a* and *slc2a3b*, Define Overlapping as Well as Distinct Expression Domains in the Zebrafish (*Danio rerio*) Central Nervous System

**DOI:** 10.3389/fnmol.2019.00199

**Published:** 2019-08-27

**Authors:** Carina G. Lechermeier, Frederic Zimmer, Teresa M. Lüffe, Klaus-Peter Lesch, Marcel Romanos, Christina Lillesaar, Carsten Drepper

**Affiliations:** ^1^Child and Adolescent Psychiatry, Center of Mental Health, University Hospital of Würzburg, Würzburg, Germany; ^2^Department of Physiological Chemistry, Biocenter, Am Hubland, University of Würzburg, Würzburg, Germany; ^3^Division of Molecular Psychiatry, Center of Mental Health, University Hospital of Würzburg, Würzburg, Germany; ^4^Laboratory of Psychiatric Neurobiology, Institute of Molecular Medicine, I.M. Sechenov First Moscow State Medical University, Moscow, Russia; ^5^Department of Neuroscience, School for Mental Health and Neuroscience (MHeNS), Maastricht University, Maastricht, Netherlands

**Keywords:** glucose transporter, nervous system, brain disorders, psychiatric disorders, brain development, GABA, GAD1

## Abstract

The transport of glucose across the cell plasma membrane is vital to most mammalian cells. The glucose transporter (GLUT; also called SLC2A) family of transmembrane solute carriers is responsible for this function *in vivo*. GLUT proteins encompass 14 different isoforms in humans with different cell type-specific expression patterns and activities. Central to glucose utilization and delivery in the brain is the neuronally expressed GLUT3. Recent research has shown an involvement of GLUT3 genetic variation or altered expression in several different brain disorders, including Huntington’s and Alzheimer’s diseases. Furthermore, *GLUT3* was identified as a potential risk gene for multiple psychiatric disorders. To study the role of GLUT3 in brain function and disease a more detailed knowledge of its expression in model organisms is needed. Zebrafish (*Danio rerio*) has in recent years gained popularity as a model organism for brain research and is now well-established for modeling psychiatric disorders. Here, we have analyzed the sequence of GLUT3 orthologs and identified two paralogous genes in the zebrafish, *slc2a3a* and *slc2a3b*. Interestingly, the Glut3b protein sequence contains a unique stretch of amino acids, which may be important for functional regulation. The *slc2a3a* transcript is detectable in the central nervous system including distinct cellular populations in telencephalon, diencephalon, mesencephalon and rhombencephalon at embryonic and larval stages. Conversely, the *slc2a3b* transcript shows a rather diffuse expression pattern at different embryonic stages and brain regions. Expression of *slc2a3a* is maintained in the adult brain and is found in the telencephalon, diencephalon, mesencephalon, cerebellum and medulla oblongata. The *slc2a3b* transcripts are present in overlapping as well as distinct regions compared to *slc2a3a*. Double *in situ* hybridizations were used to demonstrate that *slc2a3a* is expressed by some GABAergic neurons at embryonic stages. This detailed description of zebrafish *slc2a3a* and *slc2a3b* expression at developmental and adult stages paves the way for further investigations of normal GLUT3 function and its role in brain disorders.

## Introduction

The transport of the monosaccharide glucose across the cell plasma membrane is vital to most mammalian cells and is especially important for brain cells (Cremer, 1964; Norberg and Siesjö, 1974). There are two classes of transport proteins mediating glucose uptake: (1) sodium-dependent glucose transporters (GLUTs), which move glucose against the concentration gradient; and (2) sodium-independent GLUTs, which move glucose along the concentration gradient. The *GLUT* family of genes was recently renamed as solute carriers 2A (*SLC2A*) and includes 14 different isoforms in humans with different cell type-specific expression patterns and functions. This family can be further subdivided into three groups. Type I contains GLUT1, 2, 3, 4 and 14. Type II consists of GLUT5, 7, 9 and 11, and type III includes GLUT6, 8, 10 and 12 (Uldry and Thorens, 2004). Each GLUT protein consists of 12 transmembrane domains, with a central hydrophilic pore as a binding site for glucose, and with both N- and C-termini located in the cytoplasm (Navale and Paranjape, 2016).

Human GLUT3, encoded by the *SLC2A3* gene, was initially cloned from a fetal skeletal muscle cell line. It shows high expression in the brain, but is also detectable in various cancer cell lines, in placenta, colon, kidney and subcutaneous fat (Kayano et al., 1988). Since expression of GLUT3 in mice is almost exclusively restricted to the brain, with strong expression in the CA region of the hippocampus, it has been termed a neuronal glucose transporter (Nagamatsu et al., 1992). In neurons, GLUT3 is predominantly located in cell processes such as axons and dendrites, and less labeling is seen in the cell bodies (Maher, 1995; Simpson et al., 2008). This potentially makes GLUT3 one of the central proteins for control of glucose delivery and utilization in the brain (Vannucci et al., 1997). Other type I family members in the brain are GLUT4, which is actively translocated to nerve terminals during neuronal activity (Ashrafi et al., 2017), and GLUT1, which is expressed in brain parenchyma and in endothelia forming the blood brain barrier (Zheng et al., 2010). The only other type I family member, GLUT14, is a primate-specific protein expressed mainly in testis, but also with detectable expression in other tissues including the brain (Wu and Freeze, 2002; Amir Shaghaghi et al., 2016, 2017).

Recent research has shown an involvement of GLUT3 genetic variation or altered expression in several different brain diseases. Its involvement in neurodegenerative diseases for instance Huntington’s disease (Vittori et al., 2014; Morea et al., 2017; Solís-Maldonado et al., 2018), Alzheimer’s disease (Liu et al., 2008; An et al., 2018; Gu et al., 2018; Griffith et al., 2019) and glioblastoma (Cosset et al., 2017) is increasingly apparent. In addition, *SLC2A3* was identified as a potential risk gene or to display aberrant expression in several psychiatric disorders such as schizophrenia (Kuzman et al., 2009; De Silva, 2011; Sullivan et al., 2018), dyslexia (Roeske et al., 2011; Skeide et al., 2015), affective disorders (Yang et al., 2009), autism (Zhao et al., 2010; O’Roak et al., 2012; Dai et al., 2017) and attention-deficit/hyperactivity disorder (ADHD; Lesch et al., 2011; Merker et al., 2017). Therefore, the involvement of GLUT3 in several brain disorders calls for more detailed knowledge about its functional role in the nervous system.

Zebrafish (*Danio rerio*) is a genetically tractable organism that has gained popularity as a vertebrate model organism due to practical advantages and well-annotated genome. Reflecting the common evolutionary origin of all vertebrate species, zebrafish furthermore shows similarities to mammals in terms of genomes, signaling pathways, overall neurodevelopmental processes and neuroanatomy. Recently, models for symptom dimensions of psychiatric disorders such as autism and schizophrenia, as well as for the neurodegenerative diseases Alzheimer’s and Parkinson’s diseases have been developed (Stewart et al., 2015; Fontana et al., 2018). Only some members of the GLUT family of proteins have been investigated in zebrafish to date and current knowledge is restricted to GLUT1, 2, 3, 10 and 12 (Tseng et al., 2009; Zheng et al., 2010; Chiarelli et al., 2011; Willaert et al., 2012; Carayannopoulos et al., 2014; Jiménez-Amilburu et al., 2015; Marín-Juez et al., 2015; Kuwabara et al., 2018). One zebrafish gene orthologous to human *SLC2A3* has been identified (*slc2a3a*, previously termed *glut3*; Tseng et al., 2009) and its expression pattern briefly described (Carayannopoulos et al., 2014). It is ubiquitously expressed at 6 and 18 hours post fertilization (hpf) and later, at 36 hpf, concentrates in the brain and spinal cord. Knockdown of *slc2a3a* is embryonic lethal at 48 hpf with severe defects in nervous system development, microcephaly and growth retardation. These phenotypes could be rescued by overexpression of zebrafish *slc2a3a* or rat *Slc2a3* mRNAs (Carayannopoulos et al., 2014). However, a more detailed expression analysis, including other developmental stages as well as consideration of the adult brain, and identification of the type of neurons expressing *slc2a3a* are missing. In addition, many zebrafish genes are present in two copies, which is thought to be due to a whole genome duplication in the early evolution of the teleost branch of fishes (Meyer and Schartl, 1999; Glasauer and Neuhauss, 2014). The paralog of *slc2a3a*, the *slc2a3b* gene, has not yet been investigated in the context of spatial expression. This information is essential to develop a zebrafish model to study GLUT3 protein function and its involvement in brain disorders. To address this, we investigated the phylogeny of the type I GLUT family of proteins and then focused our analysis on the two GLUT3 paralogs of zebrafish encoded by the *slc2a3a* and *slc3a2b* genes. We characterized the developmental as well as the adult expression of *slc2a3a* and *slc2a3b* both during development and in adult animals using RNA *in situ* hybridization and identified the brain regions and a subset of neurons with detectable levels of the transcripts.

## Materials and Methods

### Animal Handling and Specimen Preparation

Zebrafish (*Danio rerio*) embryos (2-cell stage to 5 dpf) and adults (8–18 months) of the AB wild-type strain were used for all experiments. Developmental stages were defined according to Kimmel et al. (1995). Animals were kept on a constant day/night cycle of 14 h light/10 h darkness at 28°C. Fertilized eggs were collected and incubated in Danieau’s solution, and pigmentation of zebrafish embryos was inhibited by addition of 0.2 mM 1-phenyl-2-thiourea to the medium. Embryos were manually dechorionated followed by fixation in 4% paraformaldehyde solution (PFA) overnight at 4°C. Adult zebrafish were sacrificed with an overdose of MS-222, decapitated, and fixed overnight in 4% PFA at 4°C. Brains were dissected and further fixed for 2–4 h in 4% PFA at room temperature. Subsequently, all specimens were extensively washed in PBS with 0.1% Tween-20 (PBS-T), dehydrated through increasing methanol (MeOH) concentrations and stored in 100% MeOH at −20^°^C until use. Sampling of animal tissue material was done *post mortem*. Husbandry and euthanasia of animals were performed according to the animal welfare regulations of the District Government of Lower Franconia, Germany.

### Sequence Analysis

**Figure 1:**
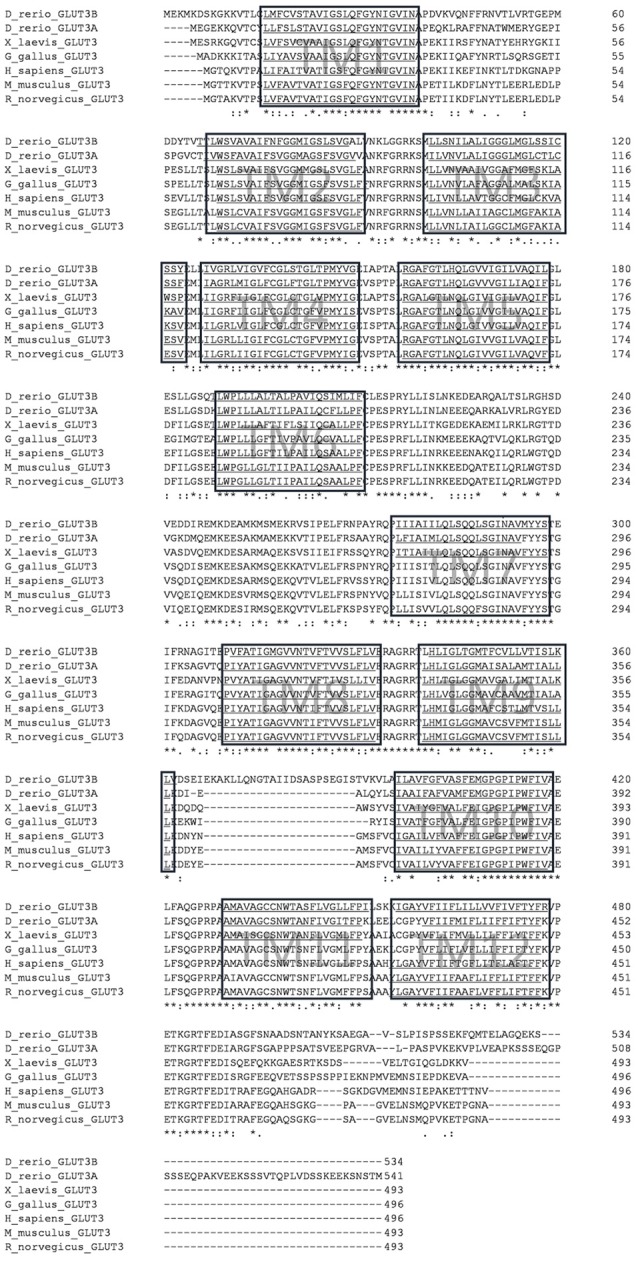
Multiple sequence alignment of glucose transporter3 (GLUT3) orthologs from selected vertebrate species. Alignment was performed with CLUSTAL Omega. Hydrophobic transmembrane (TM) regions as predicted by the TMMHM algorithm are underlined and boxed. Note the additional sequence stretch between transmembrane domain 9 and 10 in zebrafish Glut3b sequence. Accession numbers of protein sequences are given in the “Materials and Methods” section.

Human GLUT3 protein sequence (NP_008862.1) was used to identify homologs in other species using BLASTP 2.9.0 (Altschul et al., 1997, 2005). Standard settings were used except for increasing the number of target sequences to 5,000 at the NCBI homepage^1^. Sequences were mined from mouse (NP_035531.3), rat (NP_058798.2), chicken (NP_990842.1), frog (NP_001079713.1) and zebrafish (NP_001002643.1 and XP_002667169.2). Additional protein sequences, which were used for the phylogenetic analysis with the phylogeny.fr tool package (Dereeper et al., 2008) can be found in Supplementary Table S1. Alignment in the phylogeny.fr package was produced with MUSCLE (Edgar, 2004) using default settings and 16 iterations. In addition, Gblocks (Castresana, 2000), PhylML (Guindon and Gascuel, 2003) and TreeDyn (Chevenet et al., 2006) were used with default settings to conduct the analysis and generate the phylogenetic tree shown in Supplementary Figure S1. The alignment for Figure 1 was generated with the CLUSTAL Omega tool kit at the EMBL homepage^2^ using the sequences mentioned in Figure 1. Prediction of the transmembrane domains was done with TMMHM at http://www.cbs.dtu.dk/services/TMHMM/ with all sequences used for the alignment with default parameters. Motif searches were performed using the motif tool searches at http://www.genome.jp/tools/motif/. RNA-seq data (White et al., 2017) was obtained and analyzed from https://www.ebi.ac.uk/gxa/experiments/E-ERAD-475/Results.

### RT-PCR

**Figure 2:**
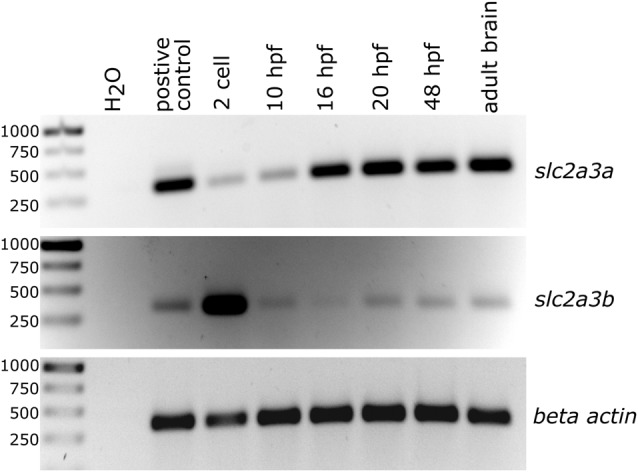
Semi-quantitative analysis of PCR products generated by reverse transcription and subsequent PCR showing temporal expression levels of *slc2a3a* and *slc2a3b* in zebrafish. Total RNA was collected at the different developmental stages as indicated. *Beta actin* (*actb1*) served as a loading control and cDNA from pooled RNA from a mixture of developmental stages as a positive control.

Total RNA was extracted from various developmental stages as indicated in Figure 2 using TRIzol^®^ Reagent (Thermo Fisher Scientific, Waltham, MA, USA) and phenol/chloroform according to manufacturer’s recommendations. Subsequently, the total RNA was treated with DNaseI (Roche) and reverse transcribed using RevertAid Reverse Transcriptase and oligo(dT) primer (Thermo Fisher Scientific, Waltham, MA, USA). cDNA was amplified by PCR using primers and cycling conditions as follows: *slc2a3a* fwd: 5′-CTATGGCTGTTGCGGGATG-3′, rev: 5′-GAGGTGGCGGATGGAGGT-3′, product size 223 bp, 68.0°C, 2.0 mM MgCl_2_, 30 s elongation, 32 cycles; *slc2a3b* fwd: 5′-GGAGAGAGCAGGGAGAAGAAC-3′, rev: 5′-CCCATCTCAAAACTAGCCACA-3′, product size: 231 bp, 60.0°C, 2.0 mM MgCl_2_, 30 s elongation, 32 cycles. cDNA synthesized from pooled total RNA collected from a mixture of developmental stages was used as a positive control. Expression of *beta actin* (*actb1*) was used as a loading control with the following primers and cycling conditions: fwd: 5′CCCAGACATCAGGGAGTGAT3′, rev: 5′TCTCTGTTGCCTTTGGGATT3′, product size: 239 bp, 53.0°C, 60 s elongation, 28 cycles.

### *In situ* Hybridization

To generate cDNA templates for synthesis of RNA probes for *in situ* hybridization (ISH), *slc2a3a* and *slc2a3b* transcripts were first amplified by RT-PCR and cloned. Total RNA was extracted from pooled embryos of different developmental stages (24–72 hpf) using TRIzol^®^ Reagent (Thermo Fisher Scientific, Waltham, MA, USA) and phenol/chloroform according to manufacturer’s recommendations. cDNA was synthesized using RevertAid Reverse Transcriptase (Thermo Fisher Scientific, Waltham, MA, USA) and oligo(dTs) primer. Primers used for amplification were as follows: *slc2a3a* (spanning exon 11 and the 3′-UTR) fwd: 5′-CTCCCAAATCATCTTCAGAACAG-3′, rev: 5′-TAGCGATTTTATTCTTGGCATAAGC-3′, product size: 1,104 bp, 64.5°C, 110 s elongation, 3.0 mM MgCl_2_; and *slc2a3b* (spanning exon 5–12) fwd: 5′-AGGAGCTTTTGGTACTCTTCAC-3′, rev: 5′-CATCTGAAACTTCTCTGATGATGG-3′, product size: 1,105 bp, 64.5°C, 3.0 mM MgCl_2_, 110 s elongation. Alternative *slc2a3b* probe (located in exon 1): fwd: 5′GGCGTCATTCTTTCACAGACAC3′, rev: 5′-GGCGCTCGTGGGTTTTATTTAG-3′, product size: 494 bp, 68.9°C, 2.0 mM MgCl_2_, 110 s elongation. The plasmid for the *gad1b* probe (Martin et al., 1998) was kindly provided by L. Bally-Cuif (Institute Pasteur).

The resulting PCR products were cloned into pCR^®^II using TA^®^ Cloning Kit Dual Promoter (Thermo Fisher Scientific, Waltham, MA, USA). Correct insertions were verified *via* Sanger sequencing (Eurofins Genomics). Linearized plasmids were purified by GenEluteTM PCR Clean-Up Kit (Sigma Aldrich, St. Louis, MO, USA) and probes were transcribed *in vitro* with either SP6 or T7 DNA polymerase using a DIG or FLUO RNA labeling Kit (Roche). Prior to use, *in situ* probes were purified with 4 M LiCl and 100% Ethanol. ISH was performed as published earlier (Thisse and Thisse, 2008). In brief, the specimens (embryos or brains) were rehydrated with PBS-T, permeabilized with Proteinase K, and post-fixed in 4% PFA for 20 min. Subsequently, the samples were pre-hybridized at 65°C in hybridization buffer (65% formamide, 5× SSC, 0.1 U/ml heparin, 5 mg/ml torula yeast RNA, 0.1% Tween 20, 9.2 mM citric acid, pH 6.0) without RNA probes for 1 h. Hybridizations with RNA probes diluted 1:100 in hybridization buffer were performed overnight at 65°C. After stringency washes at 65°C, embryos were directly processed for anti-DIG immunolabeling, while the adult brains were first embedded in 3% agarose in PBS and cut into 80 μm thick transverse sections on a vibratome (Vibratome Series 1000 Sectioning System). For anti-DIG immunolabeling all specimens (embryos and adult brain sections in agarose) were incubated in blocking buffer (PBS-T with 2% normal sheep serum and 2 mg/ml bovine serum albumin), and then for 2 h at room temperature or overnight at 4°C with sheep anti-digoxigenin-AP Fab fragments (Roche) conjugated with alkaline phosphatase (diluted 1:5,000 in blocking buffer). Alkaline phosphatase activity was detected with a nitro blue tetrazolium/5-bromo-4-chloro-3-indolyl-phosphate (NBT/BCIP) solution (Roche) diluted in fresh NTMT buffer (100 mM NaCl, 100 mM Tris-HCl, 50 mM MgCl_2_, 0.1% Tween 20). The enzymatic color reaction was stopped with PBS-T washes followed by post-fixation in 4% PFA. For double ISH, embryos were first incubated overnight at 4°C with anti-fluorescein-AP Fab fragments (Roche) diluted 1:2,000 in blocking buffer, washed with Tris buffer (0.1 M Tris-HCl, pH 8.2), followed by detection of alkaline phosphatase activity with Fast Red TR/Naphthol AS-MX Phosphate (4-Chloro-2-methylbenzenediazonium/3-Hydroxy-2-naphthoic acid 2,4-dimethylanilide phosphate) tablets (Sigma). Color reaction was stopped by washing with PBS-T. Probes were detached by incubating in PBS-T for 2 h at 68°C. New blocking, subsequent DIG probe detection and fixation were performed as described above. The specimens were finally stored and mounted in 80% glycerol. For cryosections of larval specimens pre-processed for ISH, the tissue was cryoprotected in 15% sucrose in PBS overnight, embedded in 7.5% gelatin mixed with 15% sucrose and snap frozen. Twenty micrometer thick transverse sections were cut on a cryostat, collected on SuperFrost plus slides, mounted in 80% glycerol and covered with a cover slip before imaging.

### Microscopy and Image Analysis

Whole mount *in situ* stained embryos, larval cryosections and adult brain sections were imaged with a Zeiss AxioPhot Microscope equipped with a Zeiss AxioCam MRc (Carl Zeiss Microscopy). Anatomical structures were defined and named according to prior work (Wulliman et al., 1996; Mueller and Wullimann, 2016). Images were adjusted for contrast and brightness with Fiji ImageJ (version 1.51n; Schindelin et al., 2012) and BioVoxxel Image Processing and Analysis Toolbox (Brocher, 2015). Figure panels were mounted and annotated using the freely available software tool Inkscape (version 0.92.2), available at https://inkscape.org/.

## Results

### The Zebrafish Genome Encodes Two Paralogous Copies of the GLUT3 Protein

To identify orthologs of the human GLUT3 protein, we performed database searches with the human protein sequence and obtained a longlist of candidate sequences. We found more than 100 homologous protein sequences from various species. A phylogenetic tree was constructed using type I GLUT sequences (accession numbers in Supplementary Table S1) exemplarily from human, mouse, chicken, *Xenopus tropicalis*, medaka, zebrafish, as well as GLUT from the nematode *Caenorhabditis elegans* and the sea squirt *Ciona intestinalis* (Supplementary Figure S1) with the phylogeny.fr tool package (Dereeper et al., 2008). Two zebrafish GLUT3 orthologs are present, encoded by the *slc2a3a* gene (previously known as *glut3* (Tseng et al., 2009; ENSDARG00000013295 on chromosome 19) and *slc2a3b* gene (ENSDARG00000037861 on chromosome 16). The corresponding protein sequences named here as Glut3a and Glut3b, respectively. The phylogenetic analysis suggests that these proteins share a common ancestor and show that both are present in medaka (*Oryzias latipes*), consistent with a teleost-specific genome duplication (Meyer and Schartl, 1999; Glasauer and Neuhauss, 2014). Compared to the human protein sequence, zebrafish Glut3a sequence identity is 63.91% and Glut3b is 59.88%. Glut3a, therefore, resembles the human version slightly more than Glut3b. Protein sequence conservation of the single-copy ortholog for mouse (82.46%), chicken (72.38%), and *Xenopus* (67.14%) are of intermediate values consistent with their phylogenetic distances. The sea squirt and nematode sequences are only distantly related and represent the phylogenetic outgroups in this case (Supplementary Figure S1). A comparison of the gene syntenies supports the suggestion that zebrafish *slc2a3a* and *slc2a3b* are paralogs and that zebrafish, medaka, mouse and human *slc2a3* genes are orthologs (Supplementary Figure S2A). By aligning selected vertebrate sequences with CLUSTAL Omega (Sievers et al., 2011), and performing sequence prediction of hydrophobic transmembrane domains with TMMHM (Sonnhammer et al., 1998), we determined that all of the chosen GLUT3 protein sequences share the same overall structural architecture of 12 transmembrane domains as indicated by the boxed regions in Figure 1. Interestingly, the zebrafish Glut3b sequence displays a 24-amino acid insertion between transmembrane domains 9 and 10 compared to zebrafish Glut3a, a feature that is unique to this protein. A comparison of the exon/intron structure of zebrafish *slc2a3a* and *slc2a3b* shows that these 24 amino acids are encoded by a unique *slc2a3b* exon (Supplementary Figure S2B). Detailed investigation of the hydrophobicity profiles of the sequences predicts that this additional sequence is located on the extracellular side of the plasma membrane (Supplementary Figure S3). The additional sequence between transmembrane domains 9 and 10 may be of functional relevance, due to the importance of the transmembrane 10 segment swing movement for transport activity or substrate affinity of GLUT3 (Deng and Yan, 2016).

### Temporal Expression Patterns of *slc2a3a* and *slc2a3b*

The presence of zebrafish *slc2a3a* and *slc2a3b* transcripts was detected using semi-quantitative RT-PCR analysis of total RNA extracts obtained at different developmental stages (Figure 2). Expression of *slc2a3a* is detectable from the 2-cell stage onwards, with strong expression levels from 16 hpf onwards. Expression of *slc2a3b* is detectable at very early stages (2-cell stage) and at 10, 20 and 48 hpf as well as in the adult brain (Figure 2). The early (2-cell stage) expression of *slc2a3a* and *slc2a3b* indicate that these genes are maternally expressed and deposited in the oocyte. Later, after the mid-blastula transition, transcripts are still detectable that shows zygotic transcription of the two genes. Our RT-PCR results are broadly in line with the recent high temporal resolution RNA-seq data analysis on gene expression during zebrafish embryonic development (White et al., 2017). In the RNA-seq data, the expression of *slc2a3a* is detectable above the selected threshold of 0.5 transcripts per million (TPM) at the segmentation 1–4 somite stage with 4 TPM, peaking at 36 TPM at larval day 4 (Supplementary Figure S4). In contrast, the expression of *slc2a3b* is generally lower (maximum of 3 TPM) and peaks at the 128-cell stage. Expression drops below the detection threshold after gastrula 50%-epiboly stage but is detectable again from larval hatching long-pec stage onwards again at a low level of 1 TPM (Supplementary Figure S4). Taken together, these data suggest a divergent temporal expression pattern of the two *slc2a3* paralogs in zebrafish.

### Spatial Embryonic Expression Patterns of *slc2a3a* and *slc2a3b*

**Figure 3:**
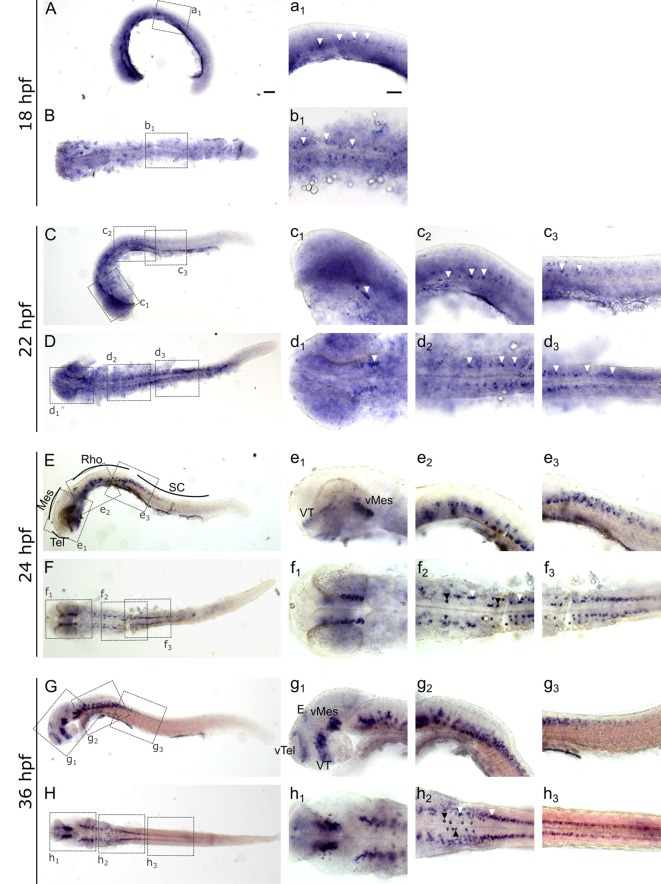
Whole mount RNA *in situ* hybridization of *slc2a3a* at early embryonic stages [18–36 hours post fertilization (hpf)]. Pictures in the left column depict alternating lateral **(A,C,E,G)** and dorsal **(B,D,F,H)** views. Anterior is to the left. Higher magnifications (a_1_–h_3_) corresponding to boxes in **(A–H)**. Arrowheads indicate examples of labeled cells or cell populations. Note staining in spinal cord, hindbrain, ventral midbrain, ventral thalamus, ventral telencephalon and epiphysis. Two bilateral rows, one medially (black arrowheads) and one laterally (white arrowheads) of positive cells are situated in the ventral hindbrain along the floor plate (f_2_ and h_2_). For abbreviations of anatomical terms see Table 1. Scale bars in **(A)**, 100 μm and pertains to **(A–H)**; scale bar in a_1_, 50 μm and pertains to a_1_–h_3_.

**Figure 4:**
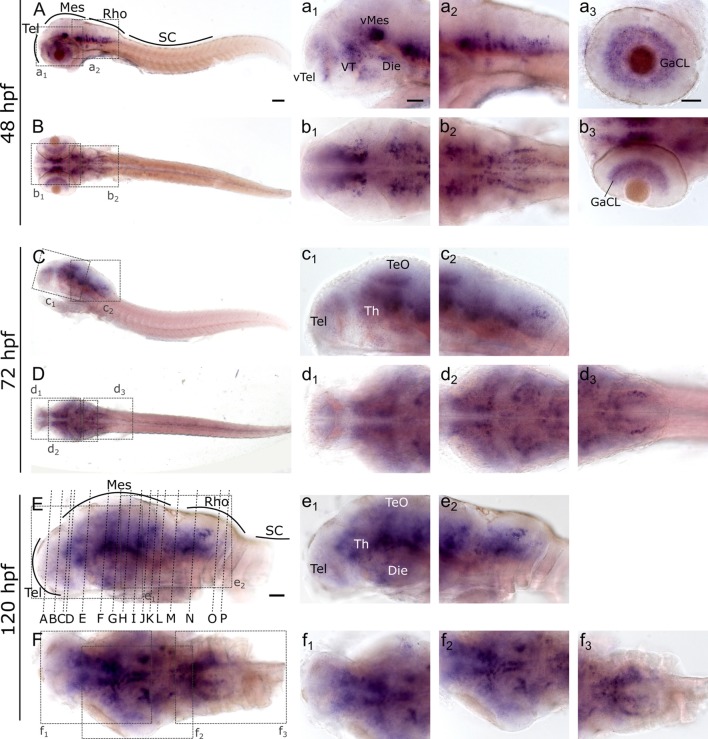
Whole mount RNA *in situ* hybridization of *slc2a3a* at late embryonic and early post-hatching stages (48–120 hpf). Pictures in the left column depict alternating lateral **(A,C,E)** and dorsal **(B,D,F)** views. Anterior is to the left. Higher magnifications (a_1_–f_3_) corresponding to boxes in **(A–F)**. Note staining in hindbrain, ventral midbrain, optic tectum, ventral thalamus, ventral telencephalon and retina. Detailed descriptions can be found in the text, for abbreviations see Table 1. Scale bar in **(A)**, 100 μm and pertains to **(A–F)**; scale bar in a_1_, 50 μm and pertains to a_1_–f_2_, d_3_, f_3_ and scale bar in a_3_, 50 μm and pertains to a_3_, b_3_.

**Figure 5:**
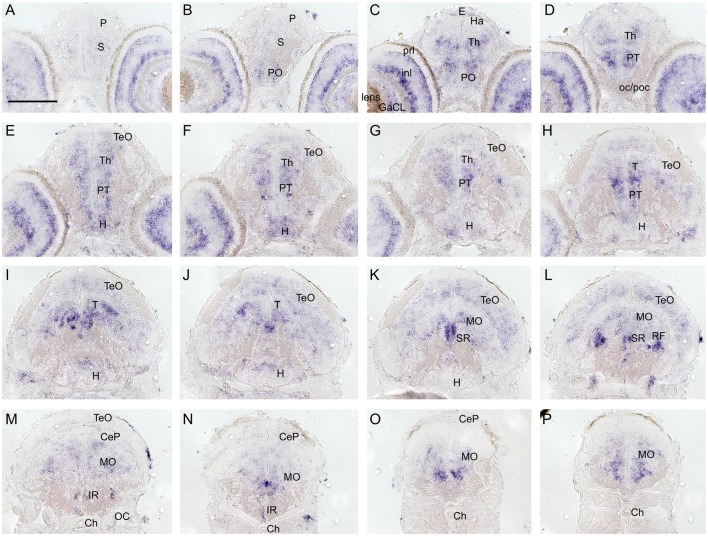
Cryosections (20 μm) of 120 hpf embryo processed for RNA *in situ* hybridization for *slc2a3a*. The sections **(A–P)** are cross sections arranged from anterior to posterior at the levels indicated in Figure 4E. Detailed descriptions can be found in the text, for abbreviations see Table 1. Scale bar, 100 μm.

To investigate and visualize the spatial distribution of *slc2a3a* and *slc2a3b* transcripts, we cloned the specific sequences spanning the open reading frame and parts of the untranslated regions of *slc2a3a* and *slc3a2b*. These were used to generate RNA probes for whole mount *in situ* hybridization of zebrafish embryos and larvae at different developmental stages. Before 16 hpf *slc2a3a* expression is diffuse and no specific cells can be identified (data not shown). From 18 hpf the first distinct positive cells are visible in the ventral hindbrain and the rostral part of the spinal cord (Figures 3A–D,a_1_–d_3_). In the hindbrain, two bilateral parallel rows of cells on either side of the floor plate are clearly detectable from at 24 hpf (Figures 3E,F). The medial row initially contains only very few cells, but the number gradually increases with developmental stage (Figures 3f_2_,h_2_). In the spinal cord, only one bilateral row of cells can be distinguished (Figures 3f_3_,h_3_). The number of positive cells along the spinal cord increases with developmental progress until 36 hpf in an anterior to posterior direction (Figures 3a_1_,c_2_,c_3_,e_2_,e_3_,g_2_,g_3_). We never detected any cells in the most caudal tip of the spinal cord, however. Longer exposure in staining solution did not lead to detection of further positive cells in the tail region (data not shown). In the ventral mesencephalon (vMes), signals from distinct cells are visible from 22 hpf (Figures 3c_1_,d_2_,f_1_). From 24 hpf onwards additional cells are stained in the ventral thalamus (VT; Figure 3e_1_), and from 36 hpf cells in the ventral telencephalon (vTel) are positive (Figures 3G,H,g_1_,h_1_). At 48 hpf a similar staining pattern is observed (Figures 4A,B,a_1_,a_2_,b_1_,b_2_), and weak expression is also present in diencephalon (Die; Figure 4a_1_) and in the ganglion cell layer (GaCL) of the retina (Figures 4a_3_,b_3_). A distinct bilateral pattern of bracket-like structures is visible in the ventral hindbrain from 48 hpf (Figure 4b_2_), which remains constant at 72 hpf (Figure 4d_3_) and 120 hpf (Figure 4f_3_). From 72 hpf onwards expression becomes visible in the optic tectum (TeO; Figures 4C,D,c_1_,c_2_,d_1_,d_2_). At 72 hpf and 120 hpf the distribution of positive cells is more complex and includes multiple populations in telencephalon, diencephalon, mesencephalon and rhombencephalon (Rho; Figures 4C–F, c_1_–f_3_). A more detailed analysis of *slc2a3a* at 120 hpf performed on transverse sections confirm the presence of transcripts in all major brain regions. The nomenclature used below is based on earlier work (Mueller and Wullimann, 2016). Faint expression is present in the subpallium (S), while stronger expression is seen in the preoptic region (PO), thalamus (Th), posterior tuberculum (PT), all along the hypothalamus (H), the optic tectum (TeO) and the medulla oblongata (MO; Figures 5A–P). Particularly strong signals are observable in the ventral tegmentum (T) and in the ventral rhombencephalon in regions overlapping with the raphe nuclei (SR and IR) as well as in the reticular formation (RF; Figures 5I–P). In the retina, expression is detectable in the retinal ganglion cell layer (GaCL) as well as in the inner nuclear layer (inl; Figure 5C).

**Table 1:** List of abbreviations of anatomical terms.

Abbrevation	Anatomical structure
AON	Anterior octaval nucleus
ATN	Anterior tuberal nucleus
CC	Cerebellar crest
CCe	Cerebellar corpus
CeP	Cerebellar plate
Ch	Chorda dorsalis
Die	Diencephalon
DIL	Diffuse nucleus of the inferior lobe
DT	Dorsal thalamus
dTel	Dorsal telencephalon
DTN	Dorsal tegmental nucleus
E	Epiphysis
EW	Edinger-Westphal nucleus
GC	Central gray
GaCL	Ganglion cell layer of retina
GrCL	Granular layer of cerebellar corpus
H	Hypothalamus
Ha	Habenula
Hc	Caudal zone of periventricular hypothalamus
Hd	Dorsal zone of periventricular hypothalamus
HTh	Hypothalamus
Hv	Ventral zone of periventricular hypothalamus
inl	Inner nuclear layer
IR	Inferior raphe
LCa	Caudal lobe of cerebellum
LH	Lateral hypothalamic nucleus
LLF	Lateral longitudinal fascicle
LVII	Facial lobe
LX	Vagal lobe
MaON	Magnocellular octaval nucleus
Mes	Mesencephalon
ML	Molecular layer of cerebellar corpus
MLF	Medial longitudinal fascicle
MO	Medulla oblongata
MON	Medial octavolateralis nucleus
NLV	Nucleus lateralis valvulae
NXm	Vagal motor nucleus
OB	Olfactory bulb
OC	Otic capsule
OT	Optic tract
oc/poc	Optic chiasm/postoptic commissure
P	Pallium
Pc	Pretectal complex
PG	Preglomerular area
PGZ	Periventricular gray zone of optic tectum
PO	Preoptic region
prl	Photoreceptor layer
PT	Posterior tuberculum
PTN	Posterior tuberal nucleus
PVO	Paraventricular organ
RF	Reticular formation
Rho	Rhombencephalon
RT	Rostral tegmental nucleus
S	Subpallium
SC	Spinal cord
SR	Superior raphe
T	Tegmentum
Tel	Telencephalon
TeO	Optic tectum
Th	Thalamus
TL	Longitudinal torus
Tla	Lateral torus
TPp	Periventricular nucleus of posterior tuberculum
TS	Semicircular torus
Va	Valvular cerebelli
Vc	Central nucleus of ventral telencephalic area
Vd	Dorsal nucleus of ventral telencephalic area
vMes	Ventral mesencephalon
VT	Ventral thalamus
vTel	Ventral telencephalon
Vv	Ventral nucleus of ventral telencephalic area

**Figure 6:**
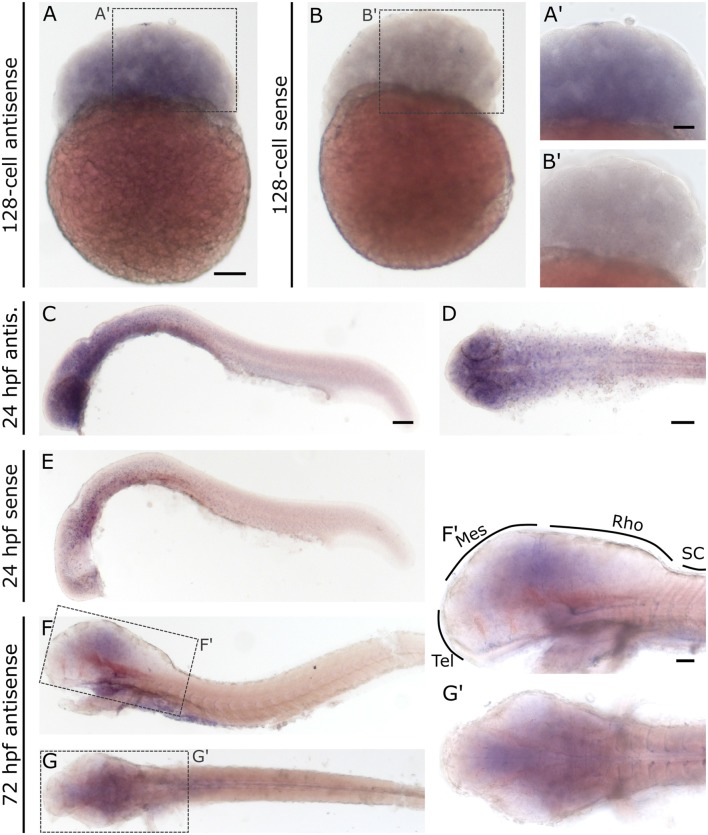
Whole mount RNA *in situ* hybridization for *slc2a3b* at blastula 128-cell, 24 hpf and 72 hpf stages. For comparison, a sense probe was included **(B,E)**. Comparison of embryos incubated with antisense **(A,A’,C,D)** and sense **(B,B’,E)** show diffuse staining only in embryos incubated with the antisense probe. Pictures (**F**; lateral) and (**G**; dorsal) show 72 hpf larvae stained with antisense probe. Boxes in **(F)** and **(G)** depict locations of high magnification pictures in **(F’,G’)**. For abbreviations see Table 1. Scale bars in **(A,C,D)**, 100 μm and pertains to **(A–G)**, respectively, scale bar in **(A’,F’)**, 50 μm and pertains to **(A’,B’,F’,G’)**, respectively.

In contrast to *slc2a3a*, *slc2a3b* displays a different and more diffuse expression pattern. Staining can be detected as early as the 128-cell stage (Figures 6A,A’). At 24 hpf staining can be seen in the brain and with decreasing intensity along the anterior-posterior-axis (Figures 6C,D). Only weak and diffuse staining in the brain is visible at 72 hpf (Figures 6F,F’,G,G’). Since the staining for *slc2a3b* is diffuse and not clearly detectable as a definite positive signal, various stages were stained in parallel with the *in situ* sense probe as a negative control (Figures 6B,B’,E). In addition, we generated an alternative *in situ* antisense probe as an additional control. This second probe exhibits a similar staining pattern as the first one used (data not shown). We, therefore, concluded that the staining for *slc2a3b* is specific, but does not highlight any distinct cell populations in contrast to *slc2a3a*.

### Expression of *slc2a3a* and *slc2a3b* in the Adult Zebrafish Brain

**Figure 7:**
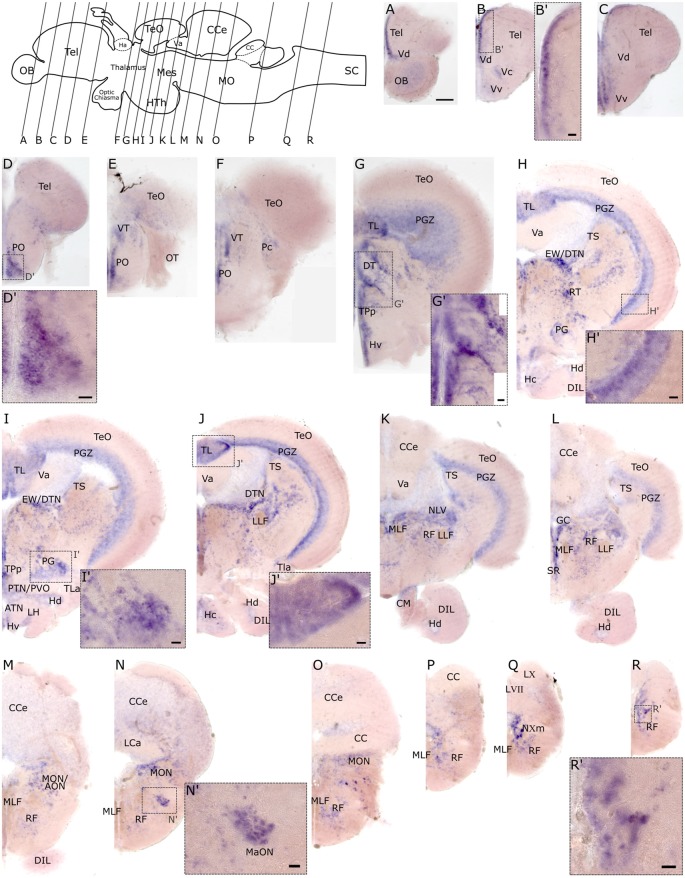
RNA *in situ* hybridization of *slc2a3a* in the adult brain. Pictures **(A–R)** show cross sections of an adult zebrafish brain (80 μm) from anterior to posterior as indicated in the scheme. **(B’–R’)** are high magnifications of boxed areas in **(B–R)**. Detailed descriptions can be found in the text, for abbreviations see Table 1. Scale bar in **(A)**, 100 μm and pertains to **(A–R)**; scale bars in **(B’–R’)**, 20 μm.

To investigate the spatial distribution of *slc2a3a* and *slc2a3b* in the adult zebrafish brain RNA *in situ* hybridization was performed on dissected brains and visualized on 80 μm thick transverse vibratome sections. Overall *slc2a3a* expression is found in all major brain compartments including telencephalon, diencephalon, mesencephalon, cerebellum and MO. The nomenclature used below is based on prior work(Wulliman et al., 1996; Yamamoto et al., 2011). In the olfactory bulb (OB), a faint staining is present in the cellular layers (Figure 7A). A stronger and more distinct signal is observed along the medial ventricular side of the dorsal telencephalon (Tel; Figures 7A–C,B’), as well as in the central (Vc), dorsal (Vd) and ventral (Vv) nuclei of the ventral telencephalon (Figures 7A–C). In the diencephalon positive cells are found in the preoptic region (PO) and ventral (VT) and dorsal (DT) thalamic nuclei (Figures 7D–G,D’,G’). Within the PT and hypothalamus transcripts, are detectable in cells located in close proximity to the ventricular systems such as the ventral (Hv), dorsal (Hd) and central (Hc) zone of the periventricular hypothalamus, periventricular nucleus of posterior tuberculum (TPp), periventricular organ (PVO) and/or posterior tuberal nucleus (PTN; Figures 7G–J). In the migrated nuclei of the PT including the preglomerular area (PG) a strong signal is also present (Figures 7H,I,I’). Of the mesencephalic structures, the periventricular gray zone (PGZ) of the optic tectum (TeO) is densely labeled (Figures 7H,H’) as well as the longitudinal torus (TL; Figures 7I,J,J’) and the ventricular side of the semicircular torus (TS; Figures 7G–L). Furthermore, several tegmental nuclei are strongly stained. These nuclei putatively include, but are not limited to, the rostral tegmental (RT), dorsal tegmental (DTN) and Edinger-Westphal (EW) nuclei, and nucleus lateralis valvulae (NLV; Figures 7H–L). In the MO, transcripts are broadly distributed, but are particularly noted in the RF (Figures 7M–R’), the magnocellular octaval nucleus (MaON; Figures 7N,N’), the region of anterior octaval nucleus (AON) and/or medial octavolateralis nucleus (MON; Figures 7M–O), and the vagal motor nucleus (NXm; Figure 7Q).

**Figure 8:**
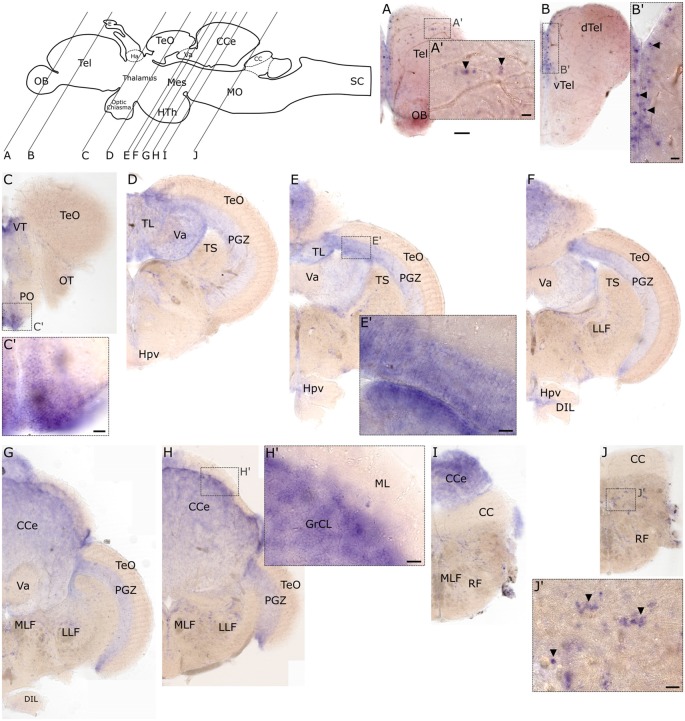
RNA *in situ* hybridization of adult expression of *slc2a3b*. Pictures show cross sections of an adult zebrafish brain (80 μm) from anterior to posterior as indicated in the scheme. Panels **(A’–J’)** are high magnifications of boxed areas in **(A–J)**. Black arrowheads in **(A’,B’,J’)** indicate example of single-positive cells. Detailed descriptions can be found in the text, for abbreviations see Table 1. Scale bar in **(A)**, 100 μm and pertains to **(A–J)**; scale bars in **(A’–J’)**, 20 μm.

Similar to the embryonic stages, *slc2a3b* exhibits less distinct expression foci in the adult brain compared to *slc2a3a*. However, some overlapping regions of expression are observed. Specifically, these include the medial ventricular side of the telencephalon (Figures 8A,B,B’), the preoptic region (PO; Figures 8C,C’), the longitudinal torus (TL; Figures 8D,E), the PGZ of optic tectum (TeO; Figures 8E,E’,F,G) and nuclei in the MO (Figures 8J,J’). *slc2a3b* is additionally visible in a few scattered cells of the dorsal telencephalon (Tel; Figures 8A,A’), the granular layer of the valvular cerebelli (Va; Figures 8D–G), and the granular cell layer (GrCL) of the cerebellar corpus (CCe; Figures 8E–I,H’). In summary, we conclude that both genes display specific staining in the adult brain. These staining patterns are mostly overlapping (i.e. PO and PGZ), but each transcript shows additional unique expression foci (i.e. many nuclei of the midbrain for *slc2a3a* and the granular cell layer of CCe for *slc2a3b*).

### Partial Co-expression of *slc2a3a* and *gad1b*

**Figure 9:**
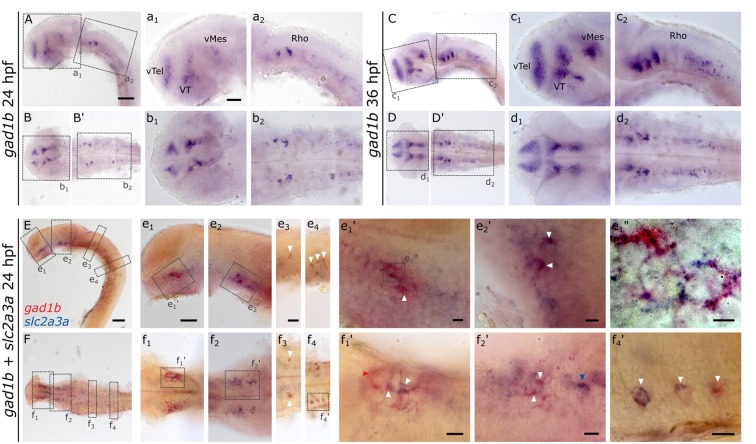
RNA *in situ* hybridization of *gad1b* and double RNA *in situ* hybridization for *slc2a3a* and *gad1b*, a marker for GABAergic neurons. For comparison, a single *in situ* hybridization for *gad1b* was performed at 24 hpf **(A,B,B’)** and 36 hpf **(C,D,D’)** old zebrafish embryos. Boxes in **(A,B,B’,C,D,D’)** depict location of high magnification pictures a_1_,a_2_,b_1_,b_2_ and c_1_, c_2_, d_1_, d_2_, respectively. In addition, a 24 hpf old zebrafish embryo was stained with *slc2a3a* DIG *in situ* probe (blue) and *gad1b* FLUO probe (red). Upper rows show lateral views **(A,C,E)** and lower rows ventral views **(B,B’,D,D’,F)**. Anterior is to the left. Boxes in **(E,F)** depict location of high magnification pictures e_1_–e_4_ and f_1_–f_4_, respectively. Boxes in e_1_ and e_2_ and f_1_, f_2_, and f_4_) represent higher magnification pictures shown in e_1_’, e_2_’ and f_1_’, f_2_’, and f_4_’. Box in e_1_’ represents location of even higher magnification shown in e_1_”. White arrows mark double-positive cells or cell populations. Red arrow marks an example of cells only positive for *gad1b* and blue arrows cells only positive for *slc2a3a*. Scale bars in **(A,E)**, 100 μm and pertains to **(A–F)**; scale bar in a_1_, 50 μm and valid for a_1_–d_2_; scale bar in e_1_, 50 μm and pertains to e_1_–e_2 _ and f_1–2_; scale bar in e_3_, 50 μm and valid for e_3_-e_4_, and f_3–4_, scale bars in e_1_’–f_4_’, e_1_’, 10 μm.

The expression pattern of *slc2a3a* strongly resembles that of *gad1b* (Mueller et al., 2006; Mueller and Guo, 2009), which is a marker for GABAergic neurons. *gad1b*, expression is detectable in the ventral telencephalon (vTel), ventral thalamus (VT), ventral mesencephalon (vMes) and rhombencephalon (Rho; Figures 9A–D,B’,D’,a_1_–d_2_), the same regions where *slc2a3a* is found (Figures 3e_1_,g_1_). To test if *slc2a3a*-expressing cells co-express *gad1b*, we performed double *in situ* hybridization. Indeed, double-positive cells can be detected at 24 hpf (Figures 9E,F). More specifically, co-expression is found in populations in the ventral telencephalon (Figures 9e_1_,f_1_), midbrain (Figures 9e_2_,f_2_) and MO (Figures 9e_3–4_,f_3–4_). At higher magnifications (Figures 9e_1_’–f_4_’) single-positive cells for *slc2a3a* (Figure 9f_2_’, blue arrow) and single-positive cells for *gad1b* (Figure 9f_1_’, red arrow) are visible, as well as double-positive (white arrows) cells for both transcripts. We noted double-positive cells at high magnification, with dyes distributed unequally in the cytoplasm (Figure 9e_1_”). We conclude, that populations of positive cells for *slc2a3a* and *gad1b* can be found in the same brain regions and that at least some cells are simultaneously expressing both transcripts. These results point towards a population of GABAergic cells in the embryonic zebrafish in which *slc2a3a* is developmentally co-expressed.

## Discussion

We have investigated the spatial and temporal expression patterns of the two zebrafish *GLUT3* orthologs *slc2a3a* and *slc2a3b*. Both transcripts display partially overlapping expression with some unique expression domains for each transcript. In addition, we were able to identify a sequence stretch of 24 amino acids, which is specific to the zebrafish Glut3b protein. Expression of *slc2a3a* is partly detectable in GABAergic cells. The detailed description of the two expression patterns now paves the way for zebrafish studies to elucidate the functional role of this important neuronal glucose transporter in neurodegenerative and psychiatric disorders.

By comparing the embryonic expression patterns of *slc2a3a* and *slc2a3b*, it became evident that *slc2a3a* shows a more prominent distribution in the developing central nervous system. We detected only a faint and early (<24 hpf) expression of *slc2a3b*, which appears to be diffuse and without clearly distinguishable single cells. In contrast, the expression of *slc2a3a* was more pronounced in several brain regions and after 18 hpf, clearly distinct single cells were visible. These results indicate that *slc2a3a* may be more critical for embryonic and larval glucose transport than *slc2a3b*. This possibility is further supported by the harsh microcephalic phenotype and growth retardation due to *slc2a3a* morpholino knockdown (Carayannopoulos et al., 2014). A similar phenotype of early embryonic death was observed in mice deficient for *Slc2a3* (Ganguly et al., 2007).

In adult brain tissue, the expression pattern is more complex. Although we detected less overall staining intensity for *slc2a3b* compared to *slc2a3a*, we were able to identify overlapping as well as unique expression domains in the brain for both transcripts. Shared expression regions were located in the telencephalon, the preoptic region, PVZ of the optic tectum and possibly some nuclei in the MO. *slc2a3b*-specific staining was detected in a few scattered cells in the parenchyma of the dorsal telencephalon and the granular cell layer of the cerebellar cortex only. Regions of overlapping expression may indicate a redundant function of *slc2a3a* and *slc2a3b*, a phenomenon that is commonly observed among paralogous genes in zebrafish, while the unique expression in some regions suggest that certain functions of the ancestral gene have been subjected to a sub-functionalization process during evolution (Postlethwait et al., 2004). Expression of GLUT3 orthologs in mammalian species, such as mouse and rat, was shown to be broadly distributed in the brain, with more distinct signals in hippocampus, cerebral cortex, striatum, and the granule cell layer of the cerebellum (Nagamatsu et al., 1992, 1993). Therefore, when using zebrafish to test GLUT3 function, particular attention has to be paid to the brain region of interest as this might impact on the interpretation of gene function in relation to disease pathology.

The embryonic staining pattern for *slc2a3a* resembled the distribution of GABAergic markers (Mueller et al., 2006, 2008). This observation was further supported by the adult expression pattern, which showed similarities with the distribution of *gad1b* (Mueller and Guo, 2009). Therefore, we hypothesized that *slc2a3a* is co-expressed by GABAergic neurons. To test this, we performed double *in situ* hybridizations with *gad1b*. These experiments demonstrated that *slc2a3a* and *gad1b* are, at least partly, expressed by the same cells during development, and thus, identify *slc2a3a* as a GABAergic marker. Currently, we cannot rule out that neuronal cells expressing other neurotransmitters, or even other non-neuronal cell-types including glial cells, are positive for *slc2a3a* expression as well. Moreover, some cells may express *slc2a3a* and/or *slc2a3b* at low levels, below the detection limit for RNA *in situ* hybridization. It will be interesting in future experiments to analyze the extent of co-expression of *slc2a3a* and *gad1b* in more detail and performing co-labeling specific for other cell types. In other organisms GLUT3 expression was shown to be detectable in multiple neuronal cell types, especially in the hippocampus of mice; this included, but was not restricted to GABAergic cells (Cembrowski et al., 2016a,b, 2018; Shah et al., 2016). Moreover, there is a debate whether GLUT3 might be expressed at low levels by astrocytes in mammalian systems, and that these cells can up-regulate their expression during certain conditions (Iwabuchi et al., 2014; Wang et al., 2016; Lee et al., 2018). The zebrafish CNS contains similar types of glia cells as the mammalian CNS, with the exception of astrocytes and radial glia (Lyons and Talbot, 2015). The distribution of *slc2a3a* and *slc2a3b* in the zebrafish brain is distinct from that of glial cells and we, therefore, conclude that *slc2a3a* and *slc2a3b* are restricted to neuronal cells, however, we cannot exclude that some cell types might alter their expression profile under specific conditions. The restricted neural expression of zebrafish *slc2a3a* to specific domains in larvae as well as adult brain raises the question which other glucose transporter plays similar roles in other brain regions. A possible candidate is GLUT2, for which a neural expression has already been described in the zebrafish brain (Marín-Juez et al., 2015). Transcripts were found to be present in liver, pronephric tubules, anterior intestine, endocrine pancreas, and importantly, in the telencephalon and hindbrain, particularly in the corpus cerebelli and MO. The precise neurotransmitter identity of the GLUT2 expressing cells still remains unknown. The zebrafish ortholog of GLUT4, which in rat is located at active synapses (Ashrafi et al., 2017), has not yet been identified. Given the fact that early embryonic depletion of *slc2a3a* leads to microcephaly, developmental delay, and embryonic death at 48 hpf in zebrafish (Carayannopoulos et al., 2014), its importance for brain maturation is evident. Similarly, depletion of *slc2a2* (*glut2)* causes developmental brain defects, which was suggested to be caused by failure to sense and regulate glucose levels in the brain (Marín-Juez et al., 2015). Considering such severe and early brain malformations upon morpholino-induced depletion in zebrafish, the importance of glucose transporters in neurodevelopment is apparent. Further research on the expression pattern of the other zebrafish GLUT proteins is needed to clarify this issue.

In a study of copy number variations in psychiatric disorders focusing on ADHD, patients were found to have an additional copy of the *SLC2A3* region (Lesch et al., 2011). The functional consequences of this for brain glucose metabolism and pathology still remains unknown, but it may be that dysregulation of brain glucose during development causes structural and/or functional alterations in the brain, which later manifests as developmental psychiatric disorders, such as ADHD (Merker et al., 2017). How might GLUT3 expression alterations be associated with brain development? A recent study found that GLUT3 is necessary for activity-induced neurite outgrowth and is central in a cascade of reactions leading to lipid production and driving neurite outgrowth (Segarra-Mondejar et al., 2018). Thus, by interfering with GLUT3 expression, and thereby with intracellular glucose levels, the maturation of disorder-relevant brain circuits might be compromised. The recent generation of a patient-specific cellular GLUT3 model might answer such molecular questions in the future (Jansch et al., 2018). Conditional ablation of *Slc2a3* in the neurons of mice led to developmental defects, less brain weight and cortical thickness. This was accompanied by functional deficits, whereas the conditional ablation in the limbic system only resulted in reductions in anxiety, spatial memories, and motor ability (Shin et al., 2018). These data indicate that GLUT3 expression variation might be associated with neurodevelopmental disorders. More research on GLUT3 functions in the brain is needed to clarify its role in psychiatric disorder mechanisms.

One additional interesting finding of our study is the identification of a unique Glut3b-specific 24-amino acid long sequence located on the extracellular side between the transmembrane domains 9 and 10. This sequence stretch is only present in the *slc2a3b* gene and is encoded entirely by a single exon (ENSDARE00000925317), exon 10 of 12 in total. In the *slc2a3a* gene structure, this exon is absent, which leads to a total 11 exons. This is the common number in other animals as well. Detailed sequence analysis in other animals revealed that only Glut3b of cave fish (*Astyanax mexicanus*) contains a somewhat related sequence stretch of 28 amino acids in a protein sequence (ENSAMXP00000020272.1) derived from genomic predictions. Both sequences contain a potential asparagine N-glycosylation motif. Intensive glycosylation of GLUT3 is a well-known phenomenon (Asano et al., 1992). Interestingly, abolishing N-glycosylation in GLUT1 was shown to decrease glucose uptake (Samih et al., 2003), indicating that this motif in Glut3b may regulate transport activity. In Fugu (*Takifugu rubripes*) and Stickleback (*Gasterosteus aculeatus*), a shorter sequence stretch of 19 amino acids with a different composition and with several proline residues is present between the transmembrane domains 9 and 10, for which no known motifs could be identified (data not shown). Further sequence searches revealed no other ortholog in animals sharing this sequence motif indicating that only some teleosts may have acquired this additional sequence during evolution. The function of this novel extracellular sequence stretch in zebrafish Glut3b is unknown. The structure of human GLUT3 has been determined as well as its conformational changes during transport activity (Deng et al., 2015). Transmembrane domains 7 and 10 are important domains performing structural movements and therefore conformational changes (Deng and Yan, 2016). Together with the potential N-glycosylation in this novel 24-amino acid extracellular domain one might speculate that it may influence substrate specificity or kinetics of zebrafish Glut3b. Further research on this topic is needed to clarify the functional relevance of teleost variations in the amino acid sequence length and composition.

## Conclusion

In conclusion, we have characterized the spatial and temporal expression pattern of zebrafish *slc2a3a* and *slc2a3b* during development as well as in the adult brain. Whereas both paralogs are expressed in the embryonic and adult nervous system, there are subtle differences in the distribution of transcripts for the two genes, indicating both redundancy and sub-functionalization between the paralogs. Interestingly, in embryonic brain we found GABAergic neurons to be positive for *slc2a3a* expression, suggesting interneuron subtype-specific expression. Furthermore, *slc2a3b* contains a previously overlooked extracellular domain, which might be important for transport activity. Taken together, this study is critical for establishing zebrafish as a model to further dissect the role of GLUT3 function in health and disease.

## Data Availability

All datasets generated for this study are included in the manuscript and/or the Supplementary Files.

## Ethics Statement

Husbandry of animals and experiments were performed according to the animal welfare regulations of the District Government of Lower Franconia, Germany.

## Author Contributions

CGL performed the experiments. FZ, CL and TL contributed to experimental support. CD and CL conducted sequence analysis. K-PL, MR, CL and CD contributed to the conception and design of the study and supervised the project. CGL, CL and CD wrote the manuscript. All authors contributed to manuscript revision, read and approved the submitted version.

## Conflict of Interest Statement

K-PL served as a speaker for Eli Lilly and received research support from Medice, and travel support from Shire, all outside the submitted work. The remaining authors declare that the research was conducted in the absence of any commercial or financial relationships that could be construed as a potential conflict of interest.

## Footnotes

^1^https://blast.ncbi.nlm.nih.gov/Blast.cgi

^2^https://www.ebi.ac.uk/Tools/msa/clustalo/
