# Supplementary material for: Transcript Analysis of Zebrafish GLUT3 Genes, slc2a3a and slc2a3b, Define Overlapping as Well as Distinct Expression Domains in the Zebrafish (Danio rerio) Central Nervous System
Source: Front Mol Neurosci. 2019 Aug 27;12:199. doi: 10.3389/fnmol.2019.00199 (PMC6718831; doi:10.3389/fnmol.2019.00199)
Supplement: Supplementary file 1 [file Data_Sheet_1.PDF]

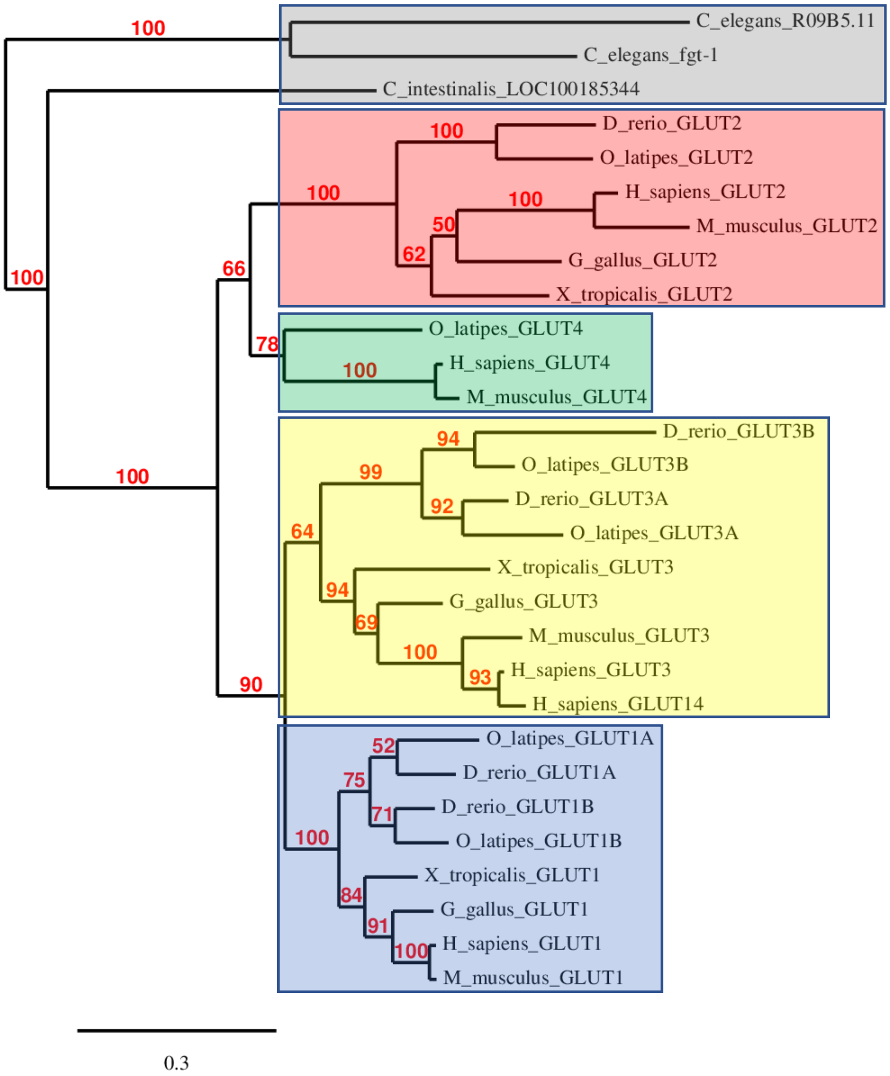

Supplementary Figure 1. Phylogenetic tree of type I GLUT protein sequences from selected species. Phylogenetic tree was constructed with the phylogeny.fr toolkit. Phylogenetic analysis confirmed that all the GLUT3 sequences share a common ancestor. Zebrafish GLUT3A sequence is closer related to the human GLUT3 than zebrafish GLUT3B. Colored boxes indicate proteins belonging to the same GLUT protein family. Blue (GLUT1), red (GLUT2), yellow (GLUT3 and GLUT14), green (GLUT4), grey (outgroup). Accession numbers can be found in Supplementary Table 1.

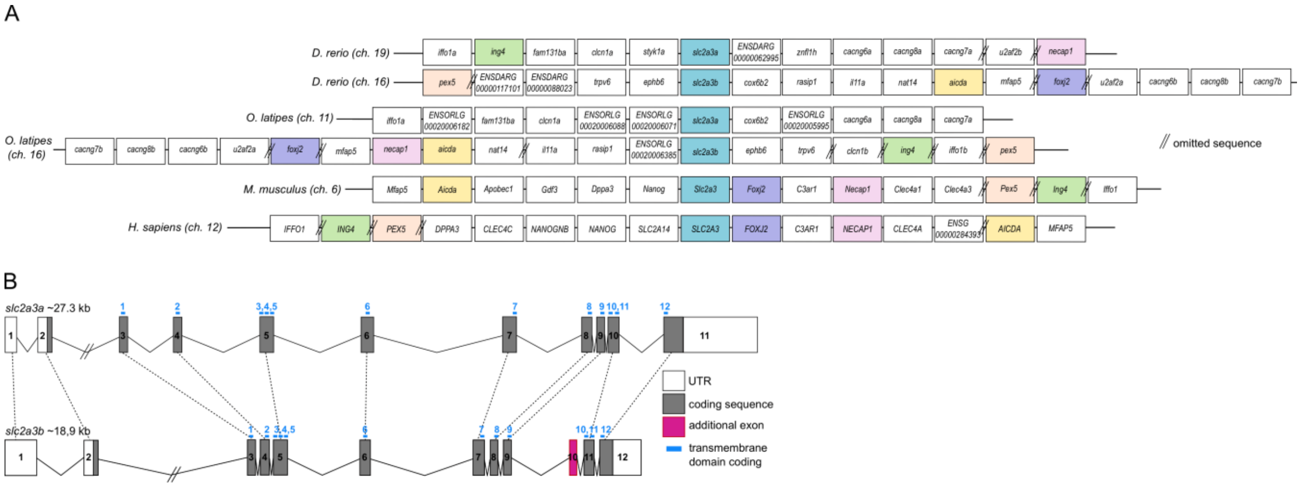

Supplementary Figure 2. A) Comparative gene syntenies centered on the loci containing *slc2a3* (turquoise) genes in two teleost fish species (*D. rerio* and *O. latipes*) and two mammalian species (*M. musculus* and *H. sapiens*). Examples of homologues are color coded. The shared synteny between teleost *slc2a3a/b* and mammalian *Slc2a3/SLC2A3* suggest that they are orthologous genes and that *slc2a3a* and *slc2a3b* are paralogous genes. The synteny was examined using genomes available on the Ensembl Genome Browser. B) Comparison of exon/intron structure of zebrafish *slc2a3a* (ENSDART00000134816.2) and *slc2a3b* (ENSDART00000019941.10). Note additional exon in *slc2a3b*. The location of the 12 transmembrane domain coding regions are indicated.

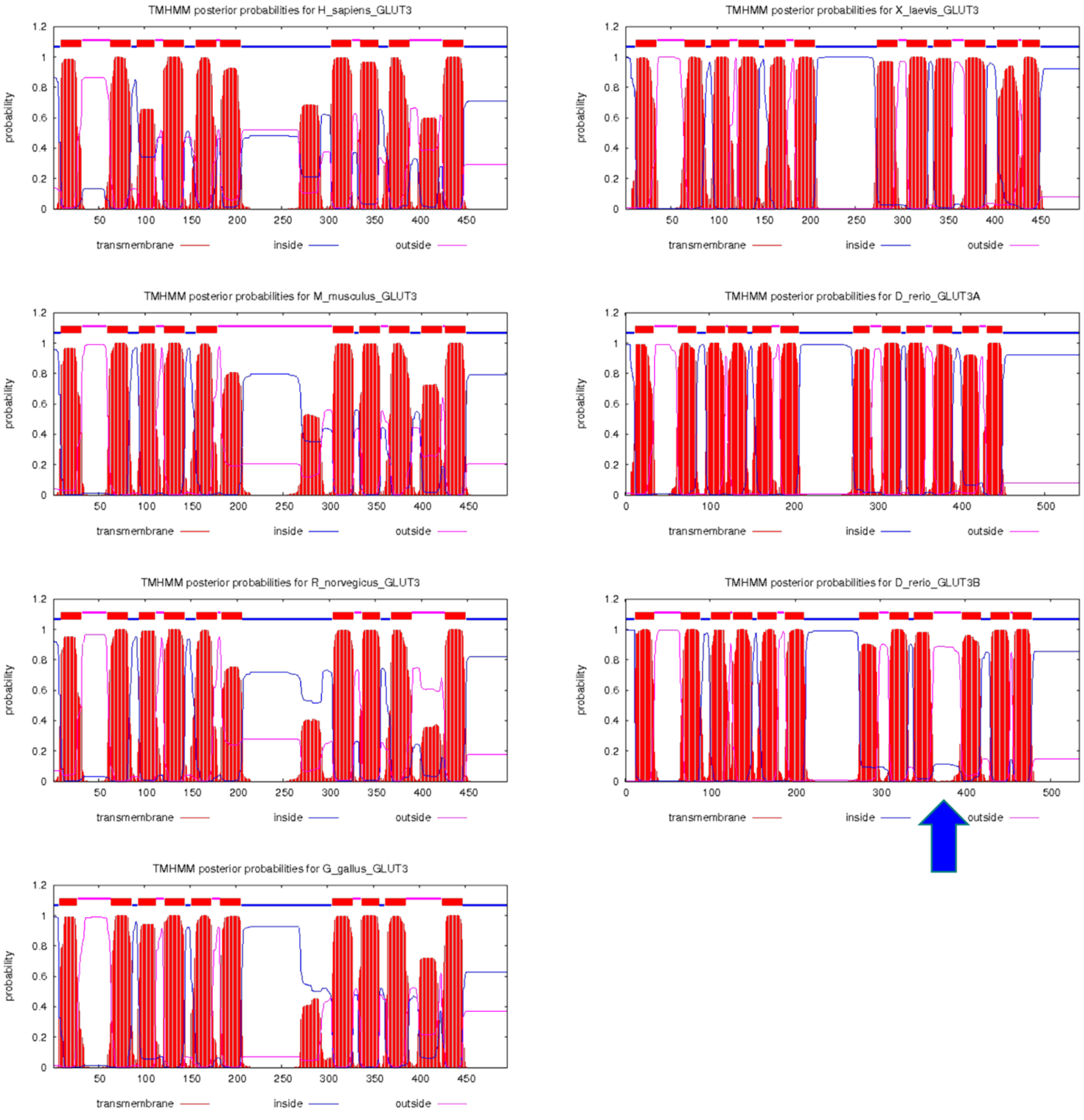

Supplementary Figure 3. Hydrophobicity profiles and identified transmembrane domains of GLUT3 orthologues from selected vertebrate species. Predictions were done with the TMMHM algorithm. Hydrophobic regions are shown in red. Note the subthreshold predictions of TM7 and TM10 in human, mouse, rat and chicken. Blue shows predicted sequence stretches in the intracellular compartment, pink indicates extracellular localization. Arrow in the zebrafish GLUT3B diagram depicts extracellular localization of the isoform-specific sequence stretch compared to the other species between TM9 and TM10.

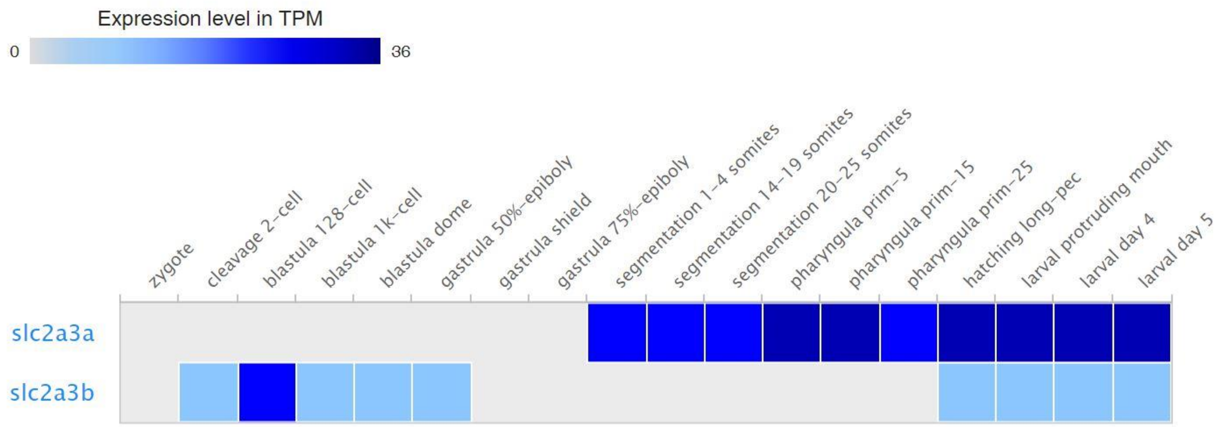

Supplementary Figure 4. Expression of the two paralogous GLUT3 genes *slc2a3a* and *slc2a3b* during zebrafish embryonic development. RNAseq data was obtained from (White et al., 2017) and visualized with the Expression Atlas Tool at (http://www.ebi.ac.uk/gxa). TKM = transcript per million.

Supplementary Table 1. Accession numbers of the protein sequences used for phylogenetic analysis in Supplementary Figure 1.

Protein group	Species	Annotated name	Accession number
**GLUT1**	Danio rerio	solute carrier family 2, facilitated glucose transporter member 1a	NP_001034897.1
	Danio rerio	solute carrier family 2, facilitated glucose transporter member 1b	tr|F1R0Q1|F1R0Q1_DANRE
	Gallus gallus	solute carrier family 2, facilitated glucose transporter member 1	NP_990540.1
	Homo sapiens	solute carrier family 2, facilitated glucose transporter member 1	NP_006507.2
	Mus musculus	solute carrier family 2, facilitated glucose transporter member 1	NP_035530.2
	Oryzias latipes	solute carrier family 2, facilitated glucose transporter member 1a	tr|H2MXW4|H2MXW4_ORYLA
	Oryzias latipes	solute carrier family 2, facilitated glucose transporter member 1b	tr|H2LTF5|H2LTF5_ORYLA
	Xenopus tropicalis	solute carrier family 2, facilitated glucose transporter member 1	tr|F6U1Z2|F6U1Z2_XENTR
**GLUT2**	Danio rerio	solute carrier family 2, facilitated glucose transporter member 2	NP_001036186.1
	Gallus gallus	solute carrier family 2, facilitated glucose transporter member 2	NP_997061.1
	Homo sapiens	solute carrier family 2, facilitated glucose transporter member 2	NP_000331.1
	Mus musculus	solute carrier family 2, facilitated glucose transporter member 2	NP_112474.2
	Oryzias latipes	solute carrier family 2, facilitated glucose transporter member 2	tr|H2M7Q1|H2M7Q1_ORYLA
	Xenopus tropicalis	solute carrier family 2, facilitated glucose transporter member 2	NP_001011453.1
**GLUT3**	Danio rerio	solute carrier family 2, facilitated glucose transporter member 3a	NP_001002643.1
	Danio rerio	solute carrier family 2, facilitated glucose transporter member 3b	XP_002667169.2
	Gallus gallus	solute carrier family 2, facilitated glucose transporter member 3	NP_990842.1
	Homo sapiens	solute carrier family 2, facilitated glucose transporter member 3	NP_008862.1
	Mus musculus	solute carrier family 2, facilitated glucose transporter member 3	NP_035531.3
	Oryzias latipes	solute carrier family 2, facilitated glucose transporter member 3a	tr|H2LNN0|H2LNN0_ORYLA
	Oryzias latipes	solute carrier family 2, facilitated glucose transporter member 3b	tr|H2M8L9|H2M8L9_ORYLA
	Xenopus tropicalis	solute carrier family 2, facilitated glucose transporter member 3	tr|F7ELF7|F7ELF7_XENTR
**GLUT4**	Homo sapiens	solute carrier family 2, facilitated glucose transporter member 4	NP_001033.1
	Mus musculus	solute carrier family 2, facilitated glucose transporter member 4	NP_033230.2
	Oryzias latipes	solute carrier family 2, facilitated glucose transporter member 4	tr|H2LPI5|H2LPI5_ORYLA
**GLUT14**	Homo sapiens	solute carrier family 2, facilitated glucose transporter member 14	NP_001273162.1
**GLUT (misc)**	Caenorhabditis elegans	facilitated glucose transporter protein 1	NP_493982.1
	Caenorhabditis elegans	facilitated glucose transporter homolog	NP_503413.2
	Ciona intestinalis	uncharacterized protein LOC100185344	tr|F6TVX8|F6TVX8_CIOIN
